# Carbon dots nanozyme for anti-inflammatory therapy *via* scavenging intracellular reactive oxygen species

**DOI:** 10.3389/fbioe.2022.943399

**Published:** 2022-08-15

**Authors:** Chen Dong, Xuehua Ma, Yi Huang, Yujie Zhang, Xiang Gao

**Affiliations:** ^1^ Department of Neurosurgery, Ningbo First Hospital, Ningbo Hospital of Zhejiang University, Ningbo, China; ^2^ CAS Key Laboratory of Magnetic Materials and Devices, Zhejiang Engineering Research Center for Biomedical Materials, Cixi Institute of Biomedical Engineering, International Cooperation Base of Biomedical Materials Technology and Application, Ningbo Institute of Materials Technology and Engineering, Chinese Academy of Sciences, Ningbo, China

**Keywords:** carbon dots nanozyme, reactive oxygen species, oxidative stress, antioxidant, anti-inflammatory

## Abstract

Developing an efficient antioxidant for anti-inflammatory therapy *via* scavenging reactive oxygen species (ROS) remains a great challenge owing to the insufficient activity and stability of traditional antioxidants. Herein, we explored and simply synthesized a biocompatible carbon dots (CDs) nanozyme with excellent scavenging activity of ROS for anti-inflammatory therapy. As expected, CDs nanozyme effectively eliminate many kinds of free radicals including ^•^OH, O_2_^•**−**^, and ABTS^+^•. Benefiting from multienzyme activities against ROS, CDs nanozyme can decrease the levels of pro-inflammatory cytokines, resulting in good anti-inflammatory effect. Taken together, this study not only sheds light on design of bioactive antioxidants but also broadens the biomedical application of CDs in the treatment of inflammation.

## Introduction

Inflammation is a component of numerous diseases and is an important immune response to a variety of factors, including pro-inflammatory cytokines such as tumor necrosis factor-*α* (TNF-*α*) (Ferrucci and Fabbri, 2018). Thus, TNF-*α* inhibitors are constantly being developed to promote anti-inflammatory therapy (Rubin et al., 2012). At present, many studies believe that the inflammation is a major feature of the tissue microenvironments (Ahmed et al., 2017). In the related pathological process, especially the initial inflammation response, has a close connection with the excessive reactive oxygen species (ROS) including superoxide radical (O_2_^•**−**^), hydrogen peroxide (H_2_O_2_), and hydroxyl radical (^•^OH) (Pei et al., 2020). The balance between generation and scavenging of ROS is precisely controlled by enzymes such as superoxide dismutase and catalase. Once the balance is disrupted, abnormal ROS levels in the inflammatory microenvironment will lead to severe cellular damage. Therefore, the effective regulation of intracellular levels of ROS is great significance to inhibit the inflammatory reactions.

In the past decade, many strategies have been employed to scavenge excessive ROS and alleviate inflammation responses, among which emerging therapeutic approaches utilizing enzymatically active nanomaterials as antioxidants have attracted more and more attention (Thakur et al., 2019; Shah et al., 2020; Wang L. et al., 2021). In recent years, many carbon-based nanomaterials with antioxidant activities have been developed to regulate abnormal ROS levels in organisms due to their efficient catalytic activities like enzymes (Das et al., 2019; Dehvari et al., 2020; Wang H. et al., 2021; Li et al., 2021; Xue et al., 2021; Ma et al., 2022). Carbon dots (CDs) nanozyme, as a “new star” among nanozymes, have potential anti-inflammatory therapeutic applications owing to their high efficiency in promoting electron transfer for scavenging ROS. Additionally, related studies have shown that since the presence of abundant groups around the *sp* ^*2*^ hybrid carbon core, CDs exhibit the characteristics of enzymatic activity that can be selectively activated (Xia et al., 2019). Chen et al. designed the tellurium-doped carbon quantum dots which can scavenge H_2_O_2_ to protect cells under ambient condition (Chen et al., 2020). Ma et al. (2022) reported the CDs with excellent superoxide dismutase enzymatic activity, which could scavenge ROS effectively (Ma et al., 2022). More recently, Wang et al. presented a novel CDs which exhibit well free radical scavenging activity and have potential to be a new highly effective antioxidant relying on the phenol-like groups (Wang X. et al., 2022). Therefore, the development of an effective therapeutic carbon-based nanozyme drug with enhanced and prolonged scavenging activities for multiple ROS is of great significance for promoting the clinical treatment progress in inflammatory disease.

**SCHEME 1:**
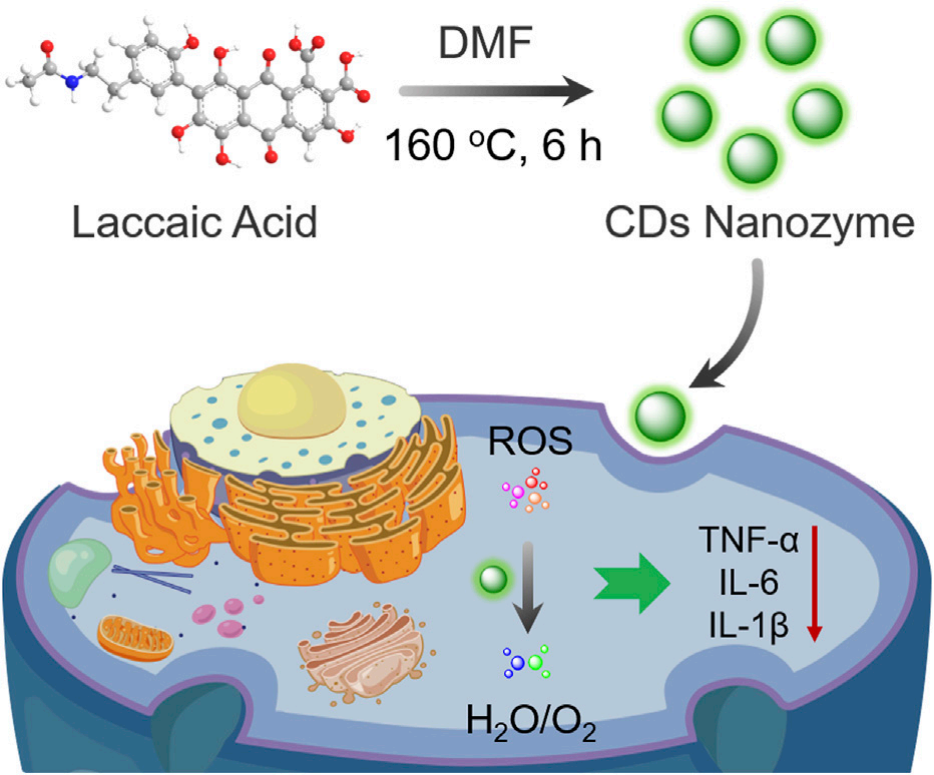
Schematic illustration of CDs nanozyme with scavenging ability against ROS for anti-inflammatory therapy.

In this study, a new type of CDs nanozyme were synthesized through a solvothermal route with the precursor laccaic acid. The presence of abundant functional groups such as carboxyl and phenolic hydroxyl groups on the surface as electron transporters endows CDs with diverse enzymatic activities. Additionally, previous works have been demonstrated that the anthraquinone structure has potential antioxidation effect (Dong et al., 2021). Noticeably, the CDs nanozyme exhibit great scavenging capability to various free radicals *in vitro*. Moreover, the therapeutic effect of CDs nanozyme by regulating the levels of pro-inflammatory cytokines in inflammatory cells were further explored (Scheme 1). Therefore, CDs nanozyme is a multifunctional nanoagents that integrates antioxidant and anti-inflammatory therapeutic functions.

## Materials and methods

### Materials

Laccaic acid was obtained from Wo Jia Biotechnology Co., Ltd. FeCl_3_·6H_2_O, *N*, *N*-Dimethylformamide, pyrogallol, and tris (hydroxymethyl) aminomethane hydrochloride were purchased from Aladdin Chemicals Co. Ltd. (Shanghai, China). Sodium chloride, ferrous sulfate, hydrogen peroxide (30 wt.%), salicylic acid, and ethanol were purchased from Sinopharm Chemical Reagent Co., Ltd. (Shanghai, China). 2,7-dichlorofluorescin diacetate (DCFH-DA), Lipopolysaccharide (LPS) and dimethyl sulfoxide were obtained from Beijing Solarbio Science & Technology Co., Ltd. Calcein-AM/PI double stain kit was obtained from Yeasen Biological Technology Co., Ltd. Cell Counting Kit-8 (CCK-8) was acquired from Saiguo Biotech Co., Ltd. HUVEC cells and RAW 264.7 cells were obtained from Shanghai Institute of Cell Biology (Shanghai, China).

### Characterization

The morphology of the CDs was recorded on a JEOL 2100 transmission electron microscope (TEM) operating at 200 kV. Fourier transform infrared (FT-IR) spectra were collected using a Nicolet 6700 spectrometer. X-ray photoelectron spectroscopy (XPS) was performed on an Axis Ultra DLD instrument. Ultraviolet-visible (UV-vis) absorption spectrum was recorded on a Lambda 950 spectrophotometer. Fluorescence spectra were performed on a HORIBA FL3-111 spectrophotometer. Cell images were captured on a Leica confocal laser scanning microscope (CLSM).

### Preparation of carbon dots nanozyme

The CDs nanozyme were prepared by a classical solvothermal method (Chen et al., 2018; Li et al., 2018). In brief, laccaic acid (0.5 g) was first dissolved in *N*, *N*-dimethylformamide, and the solution was transferred into para polyphenyl -lined autoclaves, followed by heating at 160°C for 6 h in an oven. The crude products were then purified by silica gel column chromatography using a mixture of ethyl acetate and methanol as eluent. Finally, the purified products were loaded into a dialysis membrane (MWCO 1000) and dialyzed for 4 days, and then freeze-drying to obtain a red powder.

### Free radicals scavenging activities

Three typical free radicals (O_2_^•**−**^, ^•^OH, and ABTS^+^•) were used to evaluate the antioxidant activities of CDs nanozyme (Smirnoff and Cumbes, 1989; Li, 2012).

### Cytotoxicity evaluation

Typical cytotoxicity as well as endogenous ROS generation were evaluated in detail according to standard methods reported in related reports by using human umbilical vein endothelial (HUVEC) cells and RAW264.7 cells as cell model.

### Intracellular reactive oxygen species scavenging activities

Detection of intracellular ROS scavenging efficiency by CDs nanozyme using commercial ROS probes (DCFH-DA). The protective effect of CDs nanozyme against H_2_O_2_-induced oxidative damage in a cell model was evaluated by live/dead cell double staining kit, combined with flow cytometry to measure intracellular ROS levels. Moreover, various intracellular markers including SOD, GSH, and MDA levels were detected by commercial assay kits.

### Anti-inflammation *in vitro*

RAW264.7 cells were incubated with various concentrations of CDs nanozyme and LPS (1 μg ml^−1^) for 24 h, respectively. The TNF-*α*, IL-1*β*, and IL-6 level were measured by commercial ELISA assay kits.

## Results and discussion

### Characterization of carbon dots nanozyme

**FIGURE 1:**
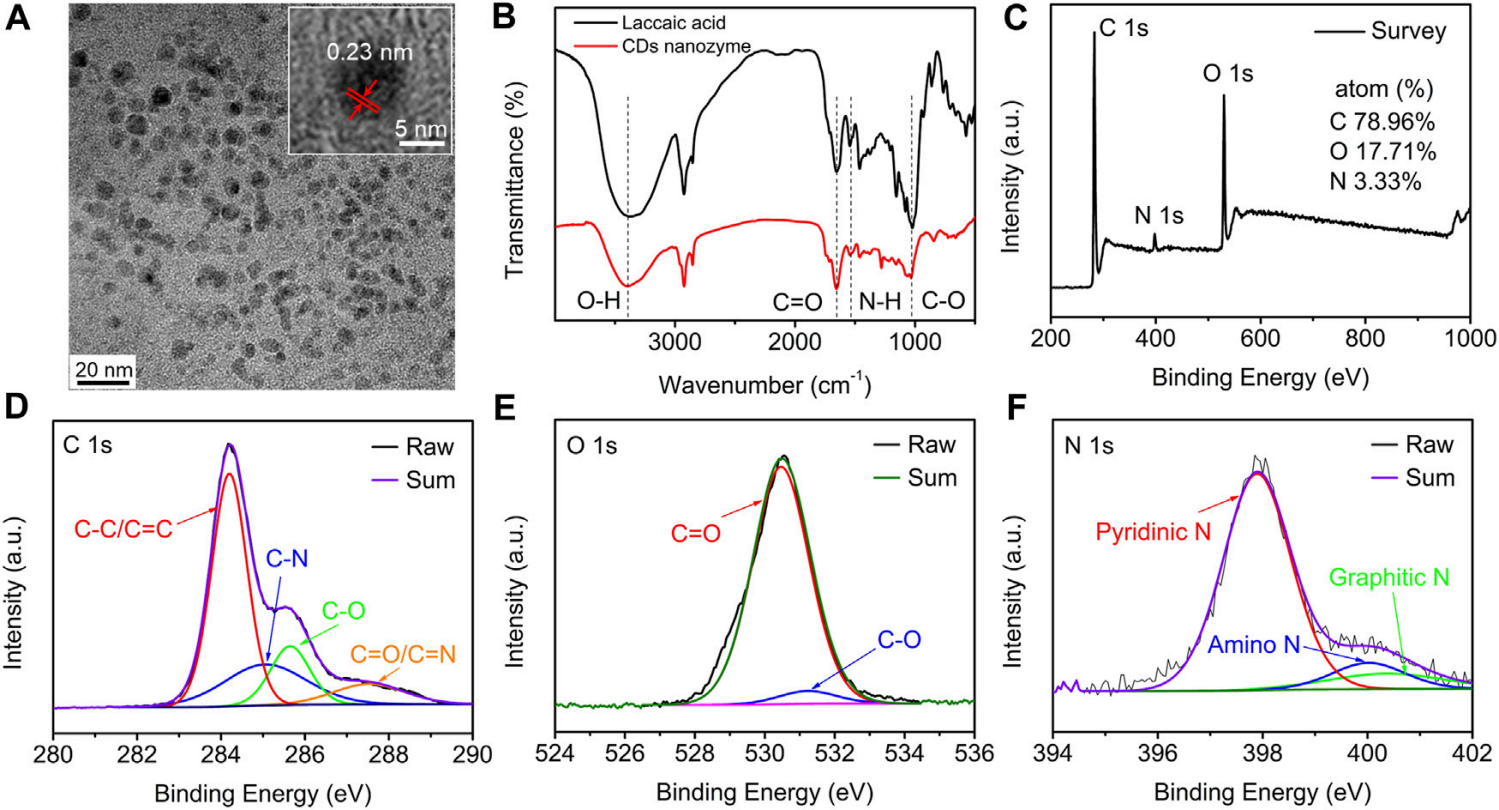
**(A)** TEM image (inset: HRTEM image) of CDs nanozyme. **(B)** FT-IR and **(C)** XPS spectra of the CDs nanozyme. **(D)** HR XPS C1s, **(E)** O1s, and **(F)** N1s spectra of CDs nanozyme and fitting results.

Transmission electron microscopy (TEM) image shows that the laccaic acid-derived CDs nanozyme are monodisperse spheroids with an average diameter of 5.51 nm (Figure 1A and Supplementary Figure S1). The average lattice spacing of 0.23 nm, which is corresponding to the (100) facet of graphite (Tang et al., 2022), can be measured in the HR-TEM image of CDs nanozyme. The chemical composition of the CDs nanozyme was determined by Fourier transform infrared (FT-IR) spectroscopy and X-ray photoelectron spectroscopy (XPS). The FT-IR spectra comparison of laccaic acid (black line) and CDs nanozyme (red line) are shown in Figure 1B. For laccaic acid, the stretching vibration peaks of −OH, C=O, N−H, and C−O are above 3391 cm^−1^, 1644 cm^−1^, 1533 cm^−1^, and1019 cm^−1^ (Zou et al., 2022). These peaks were also observed for CDs nanozyme, suggesting that CDs nanozyme inherited the natural properties of the raw materials during the preparation process. As shown in Figure 1C, the CDs nanozyme mainly consist of C (284.6 eV, at.% = 78.96), O (530.5 eV, at.% = 17.71), and N (398.5 eV, at.% = 3.33) elements. In Figure 1D, the high-resolution C 1s spectrum reveals three peaks at 284.2, 285.1, 285.7, and 287.6 eV assigned to C−C/C=C, C−N, C−O, and C=O/C=N, respectively (Dai et al., 2022). The HR XPS O 1s spectrum has two peaks, which are attributed to C=O (530.5 eV) and C−O (531.3 eV) bonds, respectively (Figure 1E) (Chen et al., 2021). The N 1s XPS spectrum (Figure 1F) can be fitted into three peaks at 397.9, 400.0, and 400.5 eV, indexing to pyridinic N, amino N, and graphitic N (Jiang et al., 2019). In addition, the absorption and fluorescence properties of CDs nanozyme were explored. As shown in Supplementary Figure S2, the absorption peaks at 268 and 368 nm are attributed to *π*−π* and *n*−π*, respectively (Guo et al., 2020; Li et al., 2020). The corresponding photoluminescence (PL) spectrum shows emission at 503 nm as shown in Supplementary Figure S3 (*λ*_ex_ = 420 nm, in methanol).

### Free radicals scavenging activities of carbon dots nanozyme

**FIGURE 2:**
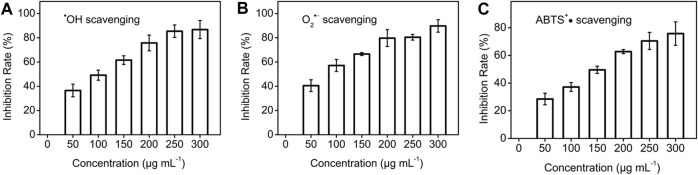
**(A)** ^•^OH, **(B)** O_2_^•**−**^, and **(C)** ABTS^+^• scavenging test of various concentration of CDs nanozyme.

The scavenging ability of CDs nanozyme to various free radicals was studied in detail. ^•^OH scavenging properties of CDs nanozyme were determined by collecting absorbance at 510 nm. As seen in Figure 2A, with the increase of the concentration, the efficiency of scavenging ^•^OH radicals reached a maximum of 86.8%. Similarly, Figure 2B shows the CDs nanozyme has a scavenging ability of 89.8% for O_2_^•**−**^ at the highest concentration. Likewise, the ABTS radical scavenging (ABTS^+^•) effects of CDs nanozyme are presented in Figure 2C. The maximum scavenging rate of ABTS^+^• free radicals by CDs nanozymes is about 75.8%.

### Biocompatibility and reactive oxygen species scavenging *in vitro*

**FIGURE 3:**
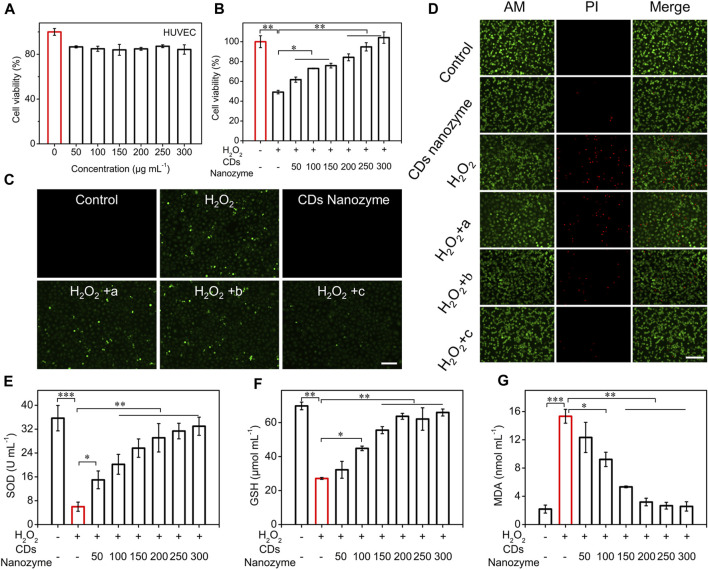
**(A)** Cell viability of HUVEC cells after treatment with various concentrations of CDs nanozyme for 24 h. **(B)** The protective effects of CDs nanozyme on H_2_O_2_-induced oxidative stress in HUVEC cells (The concentration of H_2_O_2_ is 300 μmol L^−1^). **(C)** Fluorescence images of HUVEC cells with ROS staining by DCFH-DA probe (a: 100 μg ml^**−1**^ CDs nanozyme; b: 200 μg ml^**−1**^ CDs nanozyme; c: 300 μg ml^**−1**^ CDs nanozyme, scale bar = 50 μm). **(D)** Fluorescence images of live and dead HUVEC cells stained with dyes, respectively (a: 100 μg ml^**−1**^ CDs nanozyme; b: 200 μg ml^**−1**^ CDs nanozyme; c: 300 μg ml^**−1**^ CDs nanozyme, scale bar = 50 μm). Effect of CDs nanozyme on **(E)** SOD, **(F)** GSH and **(G)** MDA in HUVEC cells.

To evaluate the potential biomedical applications of CDs nanozyme, we performed cytotoxicity studies to investigate their biocompatibility *in vitro*. The conventional CCK-8 method was used to determine the cell viability of HUVEC cells treated with CDs nanozyme for 24 h. The results show that the survival rate of HUVEC cells still keep more than 80% even with the concentration of CDs nanozyme reaching to 300 μg ml^−1^, which agrees well with previous investigations (Figure 3A) (Kuang et al., 2020). To explore the protective effect of CDs nanozyme on HUVEC cells, oxidative stress model was constructed with H_2_O_2_ (Figure 3B) (Miao et al., 2020). HUVEC cells were cultured with H_2_O_2_, cell viability decreased rapidly. However, the cell viability significantly enhanced after introducing CDs nanozyme to the system. When the concentration of CDs nanozyme was continuously increased, the cell viability continued to recover, indicating that intracellular ROS were effectively scavenged.

CDs nanozyme protect cells by regulating the level of intracellular ROS (Figure 3C). Using commercially available ROS detection probes, ROS levels were assessed during incubation. Commercially available ROS detection probe (2′, 7′-dichlorodihydrofluorescein diacetate, DCFH-DA) was used to assess ROS levels during incubation. After adding a certain concentration of H_2_O_2_ (300 μmol L^−1^) to the HUVEC cell culture medium, strong green fluorescence could be clearly observed while no fluorescence was observed in the control group. Significantly, as the concentration of CDs nanozyme continued to increased, the intracellular fluorescence intensity further reduced. Subsequently, the protective effect of CDs nanozyme against H_2_O_2_-induced oxidative stress demage by staining with live/dead cell double staining kit (calcein-AM and propidium iodide dyes) was evaluated. As can be seen from Figure 3D, H_2_O_2_ could induce most cell death after incubation, while the addition of CDs nanozyme did not cause significant cell death. Moreover, flow cytometry was used to determine the level of ROS produced by HUVEC cells in control and sample groups (Supplementary Figure S4). The result showed that the cells cultured only with H_2_O_2_ had a strong fluorescence signal, while the cells cultured with H_2_O_2_ and CDs nanozyme emit the lowest fluorescence intensity (Lin et al., 2019). The above results confirm that CDs nanozyme exhibit excellent ROS scavenging activity to protect cells from oxidative stress damage.

In addition, the effects of CDs nanozyme on superoxide dismutase (SOD), glutathione (GSH), and malondialdehyde (MDA) levels in HUVEC cells was evaluated. As shown in Figure 3E, the level of intracellular SOD was significantly decreased after the introduction of H_2_O_2_, while the level of SOD gradually increased after adding CDs nanozyme. The content of GSH in cells can be used as another important factor to measure the level of intracellular ROS. As shown in Figure 3F, the level of GSH in cells decreased to about 25% under H_2_O_2_ stimulation, while the GSH content gradually increased after the addition of CDs nanozyme. Moreover, CDs nanozyme can efficiently inhibited H_2_O_2_-induced elevation of MDA activity (Figure 3G). The above results suggest that CD nanozyme can act as an excellent ROS scavenger.

### Anti-inflammatory effect of carbon dots nanozyme *in vitro*

**FIGURE 4:**
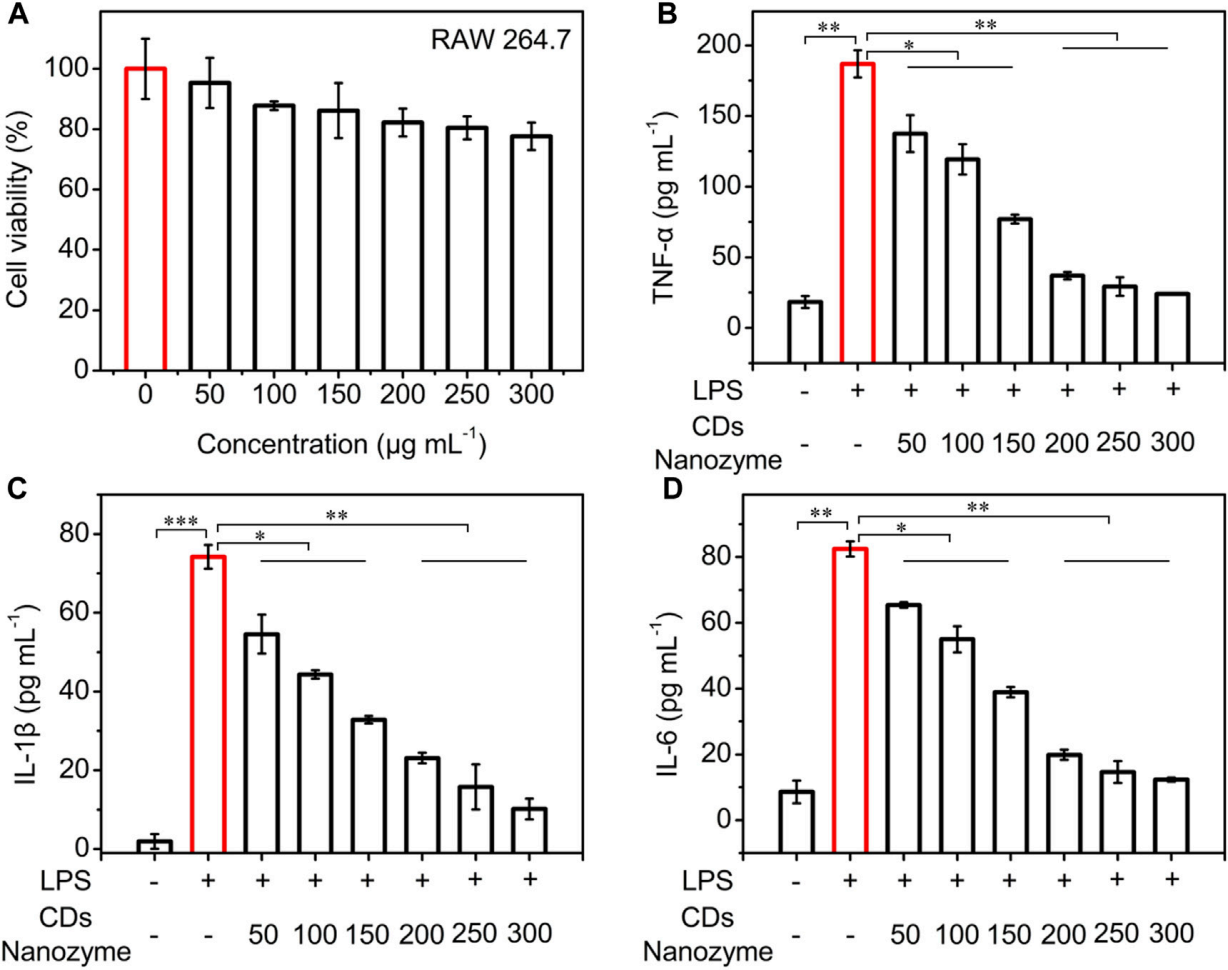
**(A)** Cell viability of RAW264.7 cells after treatment with various concentrations of CDs nanozyme for 24 h. **(B)** The TNF-*α*, **(C)** IL-1β, and **(D)** IL-6 level of the supernatants of RAW264.7 macrophages after various treatments. The concentration of LPS is 1 μg ml^−1^.

Inspired by their good biocompatibility and ROS scavenging efficiency, the anti-inflammation function of CDs nanozyme was evaluated *in vitro*. As shown in Figure 4A, the survival rate of RAW264.7 cells remained high viability even when the CDs nanozyme were cultured at relatively concentration reaching to 250 μg ml^−1^. To verify whether CDs nanozyme exhibit anti-inflammatory ability, RAW264.7 cells were treated with lipopolysaccharide (LPS) to construct a classic cellular inflammation model (Da Silva et al., 2019; Wang Z. et al., 2022; Chen et al., 2022; Kong et al., 2022). As shown in Figure 4B, the tumor necrosis factor-*α* (TNF-*α*) level of CDs nanozyme + LPS (1 μg ml^−1^) group decreased significantly compared with that of LPS group. Likewise, introduction of CDs nanozyme significantly reduced interleukin-1 beta (IL-1*β*) levels in inflammatory cell model as shown in Figure 4C. Moreover, CDs nanozyme can effectively inhibit the increase of interleukin-6 (IL-6) levels induced by LPS (Figure 4D). Subsequently, western blot results showed that CDs nanozyme upregulate the expression of P53 and Bcl-2 in HUVEC cells after 24 h of exposure (Supplementary Figure S5). In general, p53 activated only when cells have undergone stress such as ROS-induced apoptosis (Li et al., 2014). The expression of p53 and Bcl-2 in HUVEC cells after treatment with CDs nanozyme further confirmed the finding that the increased cellular level of ROS was responsible for cell death through apoptosis. The above results indicate that CDs nanozyme with ROS scavenging ability can simultaneously downregulate the levels of inflammatory cytokines (TNF-*α*, IL-1*β*, and IL-6) due to the excellent electron donor and electron acceptor properties, thereby protect the normal cells from oxidative stress damage. Moreover, CDs nanozyme inherit antioxidant active groups such as benzoquinone in the raw materials, which is also an important reason for its oxidative stress and anti-inflammatory activities.

## Conclusion

In summary, we succeeded in synthesizing a new type of CDs nanozyme by a solvothermal method. The CDs nanozyme exhibited excellent scavenging activity against various free radicals *in vitro*. Benefiting from this, CDs nanozyme can decrease the levels of pro-inflammatory cytokines (TNF-α, IL-1β, IL-6) for the efficiency treatment of inflammatory diseases. The present study indicates that CDs nanozyme can effectively inhibit oxidative stress damage through enzyme-like activity for anti-inflammatory therapy.

## Data Availability

The original contributions presented in the study are included in the article/Supplementary Material, further inquiries can be directed to the corresponding authors.
