# Supplementary material for: Carbon dots nanozyme for anti-inflammatory therapy via scavenging intracellular reactive oxygen species
Source: Front Bioeng Biotechnol. 2022 Aug 15;10:943399. doi: 10.3389/fbioe.2022.943399 (PMC9420844; doi:10.3389/fbioe.2022.943399)
Supplement: Supplementary file 1 [file DataSheet1.docx]

**SUPPLEMENTARY DATA**

**Carbon Dots Nanozyme for Anti-Inflammatory Therapy via Scavenging Intracellular Reactive Oxygen Species**

***Chen Dong^1,2^, Xuehua Ma^2*^, Yi Huang^1^, Yujie Zhang^2^, Xiang Gao^1*^***

*^1^Department of Neurosurgery, Ningbo First Hospital, Ningbo Hospital of Zhejiang University, Ningbo, China, ^2^CAS Key Laboratory of Magnetic Materials and Devices, Zhejiang Engineering Research Center for Biomedical Materials, Cixi Institute of Biomedical Engineering, International Cooperation Base of Biomedical Materials Technology and Application, Ningbo Institute of Materials Technology and Engineering, Chinese Academy of Sciences, Ningbo, China*

*****Corresponding author.

E-mail address: maxh@nimte.ac.cn, [qinyuecui@163.com](mailto:qinyuecui@163.com)


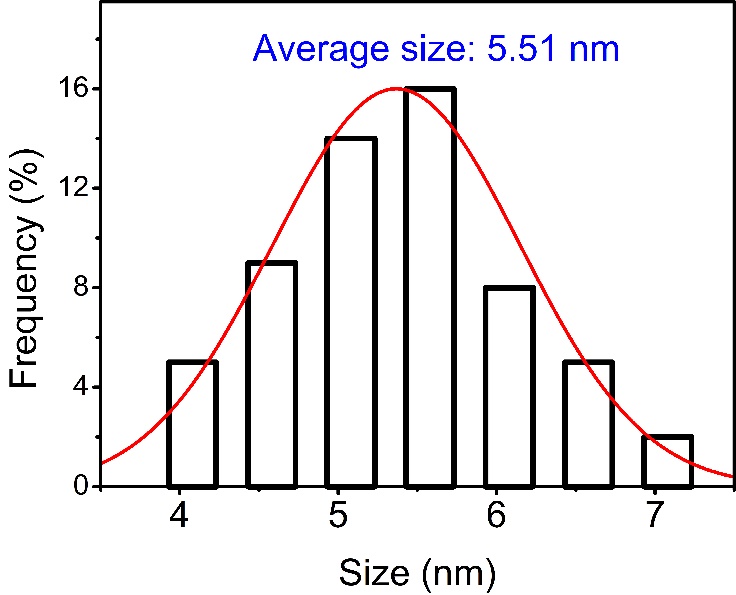


**Supple. Figure 1** The size distribution of CDs nanozyme.


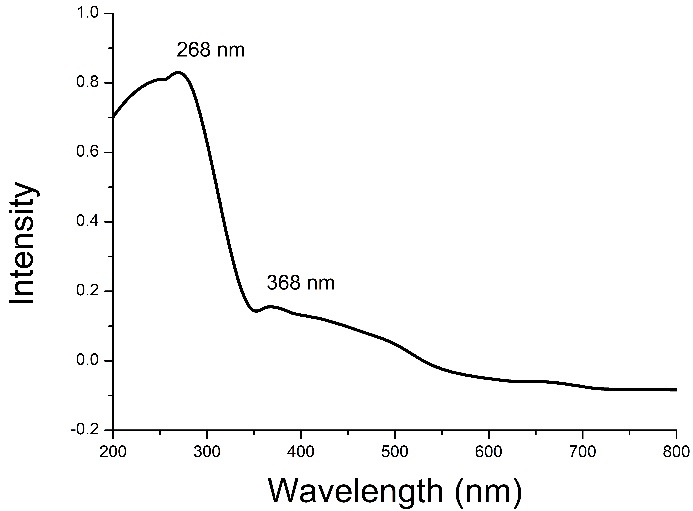


**Supple. Figure 2** UV-vis absorption spectrum of CDs nanozyme.


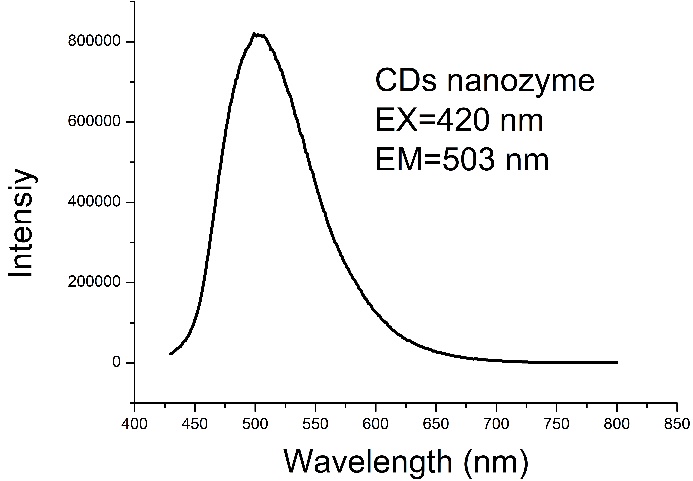


**Supple. Figure 3** PL spectrum of CDs nanozyme.


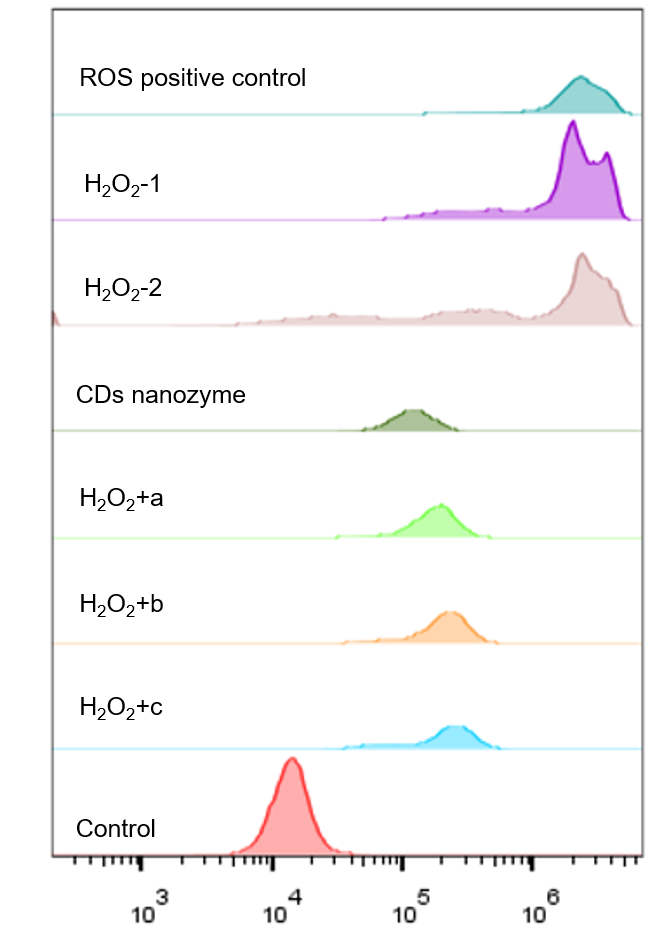


**Supple. Figure 4** The ROS level was determined by flow cytometry with DCFH-DA Dye.


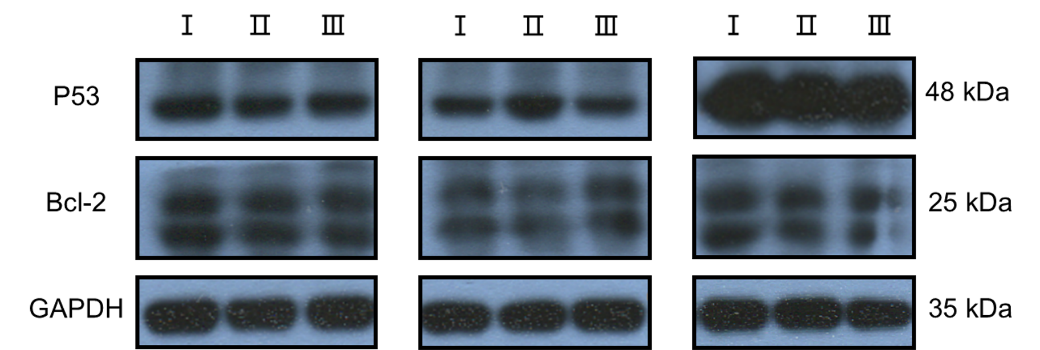


**Supple. Figure 5** Protein expression in HUVEC cells after CDs nanozyme treatment was analyzed using western blot. GAPDH as internal control (Ⅰ: control; Ⅱ: 100 μg mL^−1^ CDs nanozyme; Ⅲ: 200 μg mL^−1^ CDs nanozyme).
